# Size, composition and distribution of health workforce in India: why, and where to invest?

**DOI:** 10.1186/s12960-021-00575-2

**Published:** 2021-03-22

**Authors:** Anup Karan, Himanshu Negandhi, Suhaib Hussain, Tomas Zapata, Dilip Mairembam, Hilde De Graeve, James Buchan, Sanjay Zodpey

**Affiliations:** 1 Indian Institute of Public Health-Delhi, Public Health Foundation of India, Plot No. 47, Sector 44, Institutional Area, Sector 32, Gurugram, Haryana 122002 India; 2 South-East Asia Regional Office, Indraprastha Estate, World Health Organization, Mahatma Gandhi Marg, Outer Ring Rd, New Delhi, Delhi 110002 India; 3 Health Systems, World Health Organization, Office of the WHO Representative To India, 537, A Wing, Nirman Bhawan, Maulana Azad Road, New Delhi, 110 011 India; 4 Faculty of Health, WHO Collaborating Centre, University of Technology, Sydney, Australia

**Keywords:** Health workforce, Human resource for health, Investment in health, India

## Abstract

**Background:**

Investment in human resources for health not only strengthens the health system, but also generates employment and contributes to economic growth. India can gain from enhanced investment in health workforce in multiple ways. This study in addition to presenting updated estimates on size and composition of health workforce, identifies areas of investment in health workforce in India.

**Methods:**

We analyzed two sources of data: (i) National Health Workforce Account (NHWA) 2018 and (ii) Periodic Labour Force Survey 2017–2018 of the National Sample Survey Office (NSSO). Using the two sources, we collated comparable estimates of different categories of health workers in India, density of health workforce and skill-mix at the all India and state levels.

**Results:**

The study estimated (from NHWA 2018) a total stock of 5.76 million health workers which included allopathic doctors (1.16 million), nurses/midwives (2.34 million), pharmacist (1.20 million), dentists (0.27 million), and traditional medical practitioner (AYUSH 0.79 million). However, the active health workforce size estimated (NSSO 2017–2018) is much lower (3.12 million) with allopathic doctors and nurses/midwives estimated as 0.80 million and 1.40 million, respectively. Stock density of doctor and nurses/midwives are 8.8 and 17.7, respectively, per 10,000 persons as per NHWA. However, active health workers’ density (estimated from NSSO) of doctor and nurses/midwives are estimated to be 6.1 and 10.6, respectively. The numbers further drop to 5.0 and 6.0, respectively, after accounting for the adequate qualifications. All these estimates are well below the WHO threshold of 44.5 doctor, nurses and midwives per 10,000 population. The results reflected highly skewed distribution of health workforce across states, rural–urban and public–private sectors. A substantial proportion of active health worker were found not adequately qualified on the one hand and on the other more than 20% of qualified health professionals are not active in labor markets.

**Conclusion:**

India needs to invest in HRH for increasing the number of active health workers and also improve the skill-mix which requires investment in professional colleges and technical education. India also needs encouraging qualified health professionals to join the labor markets and additional trainings and skill building for already working but inadequately qualified health workers.

**Supplementary Information:**

The online version contains supplementary material available at 10.1186/s12960-021-00575-2.

## Introduction

Human resources for health (HRH) are a core building block of health systems [1]. The High-Level Commission on Health Employment and Economic Growth (ComHEEG) [2] emphasized that a targeted investment in health workforce promotes economic growth through range of pathways such as enhanced productivity and output, social protection and cohesion, social justice, innovation and health security. Investment in health workforce is a driver of progress towards several Sustainable Development Goals (SDGs) [2–4]. This aligns with the *Global Strategy on Human resources for Health: Workforce 2030* Report, which notes that adequate investment in health workforce along with availability, accessibility, acceptability and coverage leads to overall social and economic development along with improvements in population health [4].

Despite this increased recognition of a central role of health workforce in attaining health outcomes and enhanced economic growth, the investment in health workforce, particularly in lower and middle-income countries (LMICs) is lower than desired levels for education and training for health workers and ensuring health worker accessibility [4, 5]. This present paper aims to identify the current challenges of HRH and the areas of investment in HRH in India.

An enhanced investment in HRH has multiple benefits with the potential for a positive impact going far beyond the health sector. Further, the impact of such investments can be maximized by improving the efficiency of HRH spending in a country [2, 4]. This requires a comprehensive analysis of health workforce situation in a country and identifying the areas of investments in health workforce. Improved health workforce information base, mapping geographical regions of workforce shortage, identifying work-load and staff distribution pattern, mapping of skill-mix and training and capacity building of health workforce are of crucial importance for investment decisions at the policy levels in most LMICs [5, 6]. For instance, recent research suggesting that investment in more diverse staff and skill-mix can result in improved quality of care, quality of life, and job satisfaction [7–10]. Women constitute a significant proportion of health workforce globally. However, concentration of women in low-profile jobs within the health sector and the related gender inequality has been a serious concern particularly in (LMICs) including India [11, 12]. Profiling of health workers by age and gender helps understanding the gender issues of health workforce and women health professionals not participating in the labor markets.

The investment case for HRH in India is exemplified by the fact that India has a very low density of health workers per 10,000 population and the distribution of health workforce across the Indian states is highly skewed [13, 14]. A recent WHO report mentions that India needs at least 1.8 million doctors, nurses and midwives to achieve the minimum threshold of 44.5 health workers per 10,000 population in 2030 [15]. Also, India’s National Health Policy (NHP) 2017 recommended strengthening existing medical education system and the development of a cadre of mid-level care providers [16]. Similarly, the *NITI Aayog*’s Strategy for “New India@75” aims at generating 1.5 million jobs in the public health sector by 2022–23 [17]. The current COVID-19 pandemic has further exposed the acute shortage of health workers in India's health system. In addition, OECD countries have benefited by the presence of Indian origin and Indian trained doctors and nurses [8], while during the COVID-19 situation the health system in India is struggling with low numbers of trained health personnel.

An enhanced investment in health workforce in India has the potential of not only strengthening the health system and improving the accessibility to health workers, but also generating employment for health professionals, associate health workers and subordinate/support staff, enhancing female labor force participation and share of formal employment in total employment [15].

Recent research [13, 18] has identified several areas of concern related to Indian health workforce. Studies have highlighted that there has been acute shortages of doctors and nurses along with low levels of skill-mix. A lack of adequate number of institutions providing training in nursing, and international migration of nurses from India are the two most prominent reasons for the shortage of trained nurses in India [22–25]. Moreover, studies have also highlighted low quality of a large share of total number of nurses in India [16, 26].

Against this background, the main research question in the present study is: what are the dimensions of HRH in India which are crucial for policy attention and enhanced investment. While doing so, the study presents an updated estimate of health workforce at disaggregated geographical regions and identify issues related to difference between health workforce estimates and the stock of health professionals registered with different councils. In addition, the study also estimates level of skill-mix at the all India and state levels. To address the gender dimension of the health workforce, the study estimated level of women participation in health workforce and presents age and gender profile of health professionals who are not active in labor market.

### HRH policy and structure of health workforce in India

Indian healthcare system is characterized by a pattern of mixed ownership (public and private) and systems of medicine (allopathic and indigenous including homeopathy, *Ayurvedic, Yoga, Unani, Siddha, *etc). [16]. India’s HRH policy is shaped by recommendations by various expert committees during the past seven decades. Taking note of acute shortages and uneven distribution of health workforce in India, most of these committees recommended to significantly increase production, maintain an adequate skill-mix of health workers and maintaining minimum level of physical infrastructure at population levels [27]. However, despite these recommendations, India continued to struggle with shortages and uneven distribution of HRH. Also, sustained under-investment in public health system led private sector to overtake public in service delivery and employment of health workforce [28]. Recent health sector reforms, particularly since the launch of the National Rural Health Mission (NRHM) in 2005, focused on strengthening public health system and emphasized on improving health worker population ratio. More recently in 2019 government of India announced three strategies to enhance supply of HRH: (i) establishing new institutions to produce health workers; (ii) expand the intake capacity of the existing medical institutions and (iii) upgrade existing district hospitals to medical college level [29]. Simultaneously, government also relaxed the norms of establishing medical colleges and nursing institutions in the private sector. All these are likely to significantly increase supply of health workers in the near future.

Healthcare services in India are offered by a varied range of professionals trained in different specialties of medicine and healthcare. The supply side information [30] on the availability of health professionals indicate that these health professionals have varied levels of educational qualifications and are registered with different councils/agencies [13, 14]. Table 1 presents categories of health professionals directly engaged in services delivery along with their levels of educational qualification and their registering agencies.

**Table 1:** Types of health professionals, their educational qualification and registering agencies

Health workers	Educational qualification	Registering agencies
Allopathic doctors (physician and surgeon)	Graduates with a bachelor’s degree in medicine/surgery or postgraduate diploma	Medical council of India
Dental practitioner	Graduates with a bachelor’s or postgraduate degree in dentistry	Dental council of India
AYUSH practitioner	Graduates with a bachelor’s or postgraduate degree in Ayurveda, Unani, Siddha, or homeopathy	Department of AYUSH/MoHFW
Nurse	Diploma in General Nursing and Midwifery (3·5 year course) or a 4-year bachelor’s degree or a 2- to 3-year postgraduate degree	Indian Nursing Council
Auxiliary nurse and midwife	A diploma in auxiliary nurse midwifery (2-year course)	Ministry of health and Family Welfare
Pharmacist	Diploma or bachelor’s degree course in pharmacy	Pharmacy council of India
Physiotherapist, diagnostic and others technician	Diploma/certificate in medical allied fields	Indian Association of Physiotherapist and Ministry of health and Family Welfare

Sources: using information from CBHI 2019 and Councils of health professionals

AYUSH (an indigenous Indian system of medicine comprising Ayurvedic, Yoga, Unani, Siddha and Homeopathic) doctors are bachelor’s or postgraduate degree holders in AYUSH. Their registering institutions are Central Council for Indian Medicine or the Central Council for Homoeopathy and are authorized to dispense medicines and conduct surgery using their respective fields of specialization. AYUSH doctors are integral part of HRH in India as their professions are recognized by a Parliament Act [13, 14]. There are also community health workers with 10 years of formal education and a short training course. The health workforce at the ground level also includes many informal medical practitioners, such as registered medical practitioners (RMPs) (including traditional birth attendants, faith healers, snakebite curers, bonesetters, etc.) with or without any formal education or skills/training. RMPs are often the first point of contact for treatment for a large proportion of population living in rural and remote areas and they may be dispensing either allopathic or traditional drugs or both as the need arises [13, 14].

## Methods

The present study used data from two main sources: (1) National Health Workforce Accounts (NHWA) on India-2018 [31] and (2) Periodic Labour Force Survey (PLFS) conducted during July 2017–June 2018 by the National Sample Survey Office (NSSO 2017–2018) [32]. In addition, information was also collected from Central Bureau of Health Intelligence (CBHI) 2019, Rural Health Statistics (2019) and population projection from the Census of India (2019) [33].

### NHWA data

The NHWA for India provides information on different categories of stock of health workers at national and state levels. The latest information available is for the year 2018. We extracted number of health professionals from NHWA for four different categories (medical doctors, dentist, nurses/midwives/auxiliary nurse and midwives (ANM), and pharmacist) at the all India and state levels for the year 2018.

### NSSO data

The sample size of PLFS 2017–2018 is 102,113 households (56,108 rural and 46,005 urban) covering 433,339 individuals (246,809 rural and 186,503 urban). The survey collected information related to the nature of occupation of workers using National Classifications of Occupation (NCO) 2004 and the National Industrial Classification (NIC) 2008. NSSO data also provide information on detailed activity status such as worker, unemployed and out of labor force, location of workers by state and rural and urban, general educational and technical educational qualifications, place of working by public and private sectors.

### Methods of estimation of health workforce

Total stock of health professionals by types of health professionals (doctors, nurses and midwives, pharmacists and traditional medicine practitioners) is directly reported in the NHWA database. We estimated size of comparable categories of health workforce from the NSSO 2017–2018, using the worker population ratio (WPR) and projected population as of January 2018. We applied the WPR at the disaggregated occupational levels estimated from NSSO 2017–2018 to the projected population as of 1 January 2018 using population projection at disaggregated levels: male and female living in rural and urban areas separately in each state. The estimates of HRH were arrived at using Eq. (1):

1 $${\text{HW}}_{aijk} = {\text{pop}}_{ijk} {\text{* WPR}}_{aijk} ,$$

where ‘${\mathrm{HW}}_{a}$’ represents health workers from categories ‘a’ (representing doctors, dentists, AYUSH, nurses and so on); ‘pop’ is the projected population as of January 2018 and *‘*${\mathrm{WPR}}_{a}$ is worker participation ratio for each category in years 2017–2018. The subscripts *i*, *j* and *k* represent gender, rural–urban and states. WPR in each category of workers was estimated using Eq. (2):

2 $${\text{WPR}}_{a} = {\raise0.7ex\hbox{${{\text{workers}}_{a} }$} \!\mathord{\left/ {\vphantom {{{\text{workers}}_{a} } {{\text{pop}}}}}\right.\kern-\nulldelimiterspace} \!\lower0.7ex\hbox{${{\text{pop}}}$}}.$$

The NSSO survey reports up to two self-reported activities of all persons based on major and short time dispensation criteria separately. We considered both activities of each individual and identified health workers on the basis of either primary or secondary status. Information on activity status and educational background of each individual was used for identifying ‘unemployed’ and ‘not in labor force’ statuses of persons with medical qualifications.

The existing NCO 2004 and NIC 2008 codes used in the 2017–2018 survey could not identify disaggregated numbers of health professionals by allopathic doctors, AYUSH doctors and dentists employed in hospital settings, although the same were identified outside the hospital setting. We applied the ratio of different health professionals outside the hospital sector on the hospital sector to arrive at the total estimate of different categories of health workers. The cross classification of NCO 2004 and NIC 2008 for identifying different categories of workers is presented in Additional file 1: Appendix Table A-I.

The two sources (NHWA and NSSO data) identify comparable categories of health professionals. However, NSSO data base does not provide NCO code for identifying ANM and pharmacists comparable to the NHWA. It is possible that a part of the total ANM number in the NSSO data may be clubbed in another category coded as ‘health associate professionals’. The pharmacist number presented in this report on the basis of NSSO data only refers to pharmacists engaged in retail trade.

### Supply side estimation

We estimated the supply of health professionals in future years up to 2030 using estimated number of seats in different medical colleges/institutions. Institutions offering health programs in 2019 were identified through Google search engine using keywords such as “health programs”, “nursing courses”, “AYUSH”, “MBBS”, “BPharma” and “allied health programs”. The search was limited to programs offered in India. Additionally, the websites of the All India Council of Technical Education, University Grants Commission, universities and institutions were also searched, and education supplements of newspapers and commercial websites were searched.

The number of seats in various health professional programs was forecasted for the period till 2030. We assumed a seat occupancy rate of 95% for medical doctors for the forecast time period. For generating the workforce estimates for each year, we added the new supply for each year to the workforce numbers in the preceding year and subtracted assumed exits from the workforce to account for mortality, retirement and migration by assuming an overall annual attrition rate of 7% every year.

Finally, we modeled scenarios according to different levels of policy intervention which was similar to that adopted by Ridoutt et al. [34].

## Results

### Size and composition of health workforce

Table 2 presents estimates of HRH, categorized by doctors, dentists, nurses/midwives and pharmacist, at the all India level using the two main sources of data. Since workers self-reported occupations in the NSSO survey and health workers may or may not have adequate qualifications, we present estimates on health workforce from NSSO with and without adequate qualifications.

**Table 2:** Size and composition of HRH in India as of 2018

HWF	NHWA (millions)	NSSO (millions)		NSSO estimate as % of NHWA	
		Total reported	Adequately qualified	Total reported	Adequately qualified
Allopathic doctor	1.16	0.80	0.66	72.7	60.0
Nurse/midwives	2.34	1.40	0.79	60.9	34.3
Pharmacist	1.19	0.25	0.21	21.0	17.6
Dentist	0.27	0.18	0.17	66.7	63.0
Traditional medicine professional/AYUSH	0.79	0.49	0.25	62.0	31.6
Health Associates/allied*	N.A	0.75	0.40	N.A	N.A
Overall	5.76	3.87	2.48	67.2	43.1

Sources: NHWA 2018; NSSO 2017–2018 and Census of India 2019

*Includes health assistants, sanitarians, dieticians and nutritionists, optometrists and opticians, dental assistants, physiotherapy associates, pharmacist assistants, occupational therapist chiropodist, masseur, etc.

NHWA reports a total stock of approximately 1.16 million allopathic doctors, 2.34 million nurses/midwives (including ANM), 1.20 million pharmacists, 0.27 million dentists, and traditional medicine professionals 0.79 million. Both the estimates (with and without adequate qualifications) from NSSO are invariably lower compared with the NHWA estimates for all the reported categories. According to NSSO, the numbers of allopathic doctors and nurses/midwives, even before adjusting for the right qualifications, are 0.80 million and 1.4 million, respectively. Estimates on pharmacist, dentist, and traditional medical practitioners from NSSO are also significantly lower as compared with those recorded in the NHWA.

The difference in the estimates from the two sources are the highest for nurses/midwives and pharmacists. For nurses/midwives categories, ANM is not recorded separately in the NSSO and may be clubbed partly with nurses/midwives and partly with health associates. For pharmacists, only pharmacists engaged in the retail trade were identifiable in the NSSO data and pharmacist assistants are clubbed in the health associate category. The NSSO-based estimates after adjusting for the mandated qualifications are further lower as 18% of health workers who self-reported as allopathic doctors and 44% of health workers engaged as nurses/midwives had no adequate qualification.

State-wise dis-aggregation of allopathic doctors and nurses reflect large concentration of stock of health professionals in a few states like Maharashtra, Tamil Nadu and Karnataka (Table 3) and active health workforce in states of Uttar Pradesh, West Bengal and Kerala (Additional file 1: Appendix Table A-II).

**Table 3:** Percentage distribution of allopathic doctors and nurse across states, 2018

	NHWA		NSSO estimates	
State	Doctors	Nurses	Doctors	Nurses
Andhra Pradesh	9.09	12.38	4.33	6.64
Assam	2.16	1.68	1.46	3.66
Bihar	3.67	0.6	7.04	1.86
Chhattisgarh	0.79	0.88	1.04	2.88
Delhi	1.93	2.4	1.58	4.30
Gujarat	6.05	5.73	3.94	4.97
Haryana	0.52	1.9	1.37	2.66
Himachal	0.28	1.09	0.14	0.51
Jammu and Kashmir	1.36	0	2.38	0.78
Jharkhand	0.53	0.27	1.46	1.59
Karnataka	11.1	9.53	4.65	4.39
Kerala	5.36	10.22	11.10	5.85
Madhya Pradesh	3.45	5.28	8.48	3.02
Maharashtra	15.67	7.02	7.49	8.78
NE States*	0.39	1.34	1.88	2.66
Odisha	2.04	4.6	1.14	2.37
Punjab	4.37	3.33	0.83	3.37
Rajasthan	3.92	10.31	2.41	5.30
Tamil Nadu	12.24	11.73	6.74	10.99
Telangana	0.45	0.51	3.93	3.97
Uttar Pradesh	7.01	4.51	13.72	9.97
Uttarakhand	0.78	0.17	0.74	1.92
West Bengal	6.51	4.54	11.39	6.75
Union Territories	0	0	0.78	0.81

Sources: NHWA 2018 and NSSO 2017–2018

*Includes north-east states of Arunachal Pradesh, Manipur, Meghalaya, Mizoram, Nagaland, Sikkim and Tripura

### Density of doctors and nurses and skill-mix

**Fig. 1:**
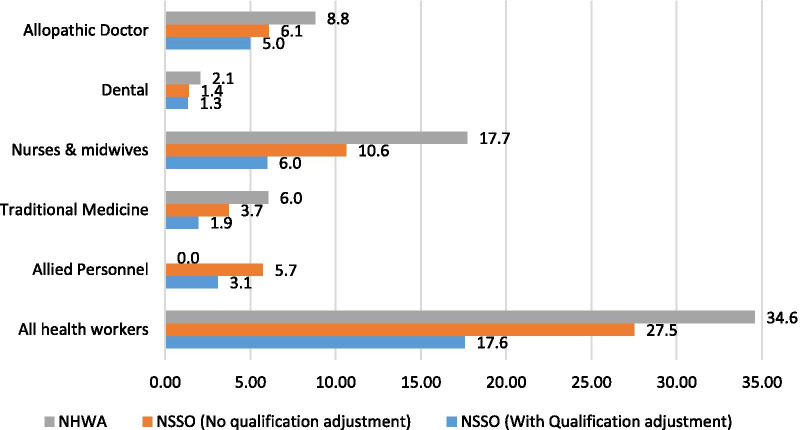
Number of health professionals/workers per 10,000 persons, 2018. Sources: estimates from NHWA 2018 and NSSO 2017–2018. Using population projection as of 1st January 2018 from Census of India 2019

At the all India level, stock density of doctor and nurses/midwives are 8.8 and 17.7, respectively, per 10,000 persons (Fig. 1). If we add total stock of dentists and traditional medicine practitioners, total stock density in India is estimated as 34.6 per 10.000 persons. However, density of active workers (as estimated from the NSSO) of doctor and nurses/midwives (without adjusting for adequate qualification) is estimated to be 6.1 and 10.6, respectively. The density further drops to 5.0 and 6.0, respectively, after adjusting for the adequate qualifications. Total active worker density is estimated to be 26.5 and 16.7, respectively, before and after adjusting for qualifications.

**Fig. 2:**
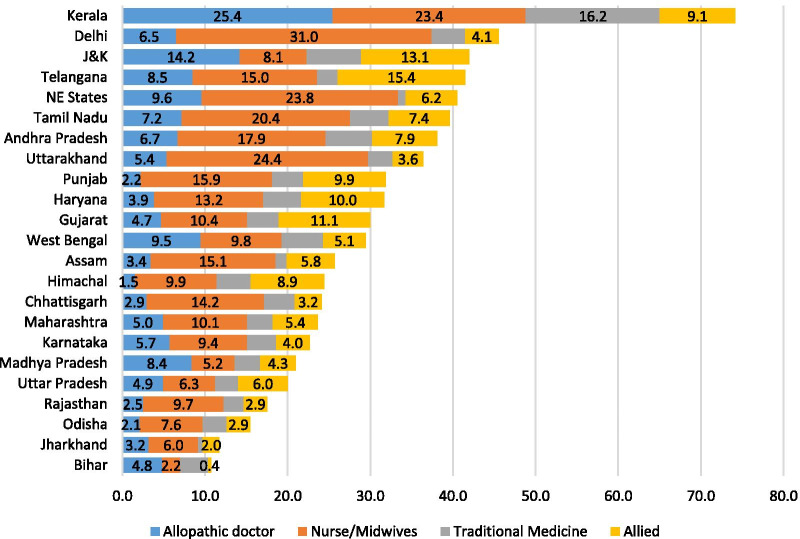
Density of health workers/professionals in states, 2018. Sources: estimates from NSSO 2017–2018. Using population projection as of 1st January 2018 from Census of India 2019

Among the states, Kerala reported the highest density of active doctor workforce (25.4), whereas Delhi had the highest density of active nurse/midwives workforce estimated from NSSO. Considering doctor and nurse workforce together, Kerala, Delhi and Tamil Nadu are on the top of the list with a great deal of variations across states (Fig. 2) (see Additional file 1: Appendix Table A-III for details).

**Fig. 3:**
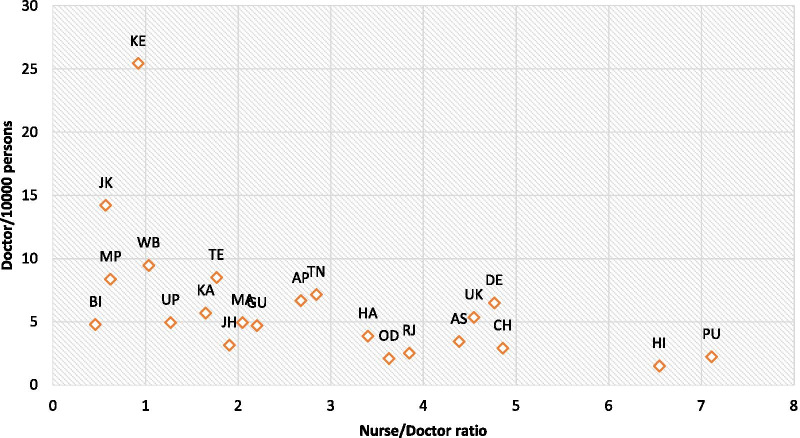
States with varied density of doctors and nurse/doctor ratio. *DE* Delhi, *HA* Haryana, *HI* Himachal Pradesh, *JK* Jammu and Kashmir, *PU* Punjab, *RJ* Rajasthan, *UK* Uttarakhand, *AS* Assam, *CH* Chhattisgarh, *MP* Madhya Pradesh, *UP* Uttar Pradesh, *BI* Bihar, *JH* Jharkhand, *WB* West Bengal, *OD* Odisha, *MA* Maharashtra, *GU* Gujarat, *AP* Andhra Pradesh, *KA* Karnataka, *KE* Kerala, *TN* Tamil Nadu, *TE* Telangana Source: estimates from NSSO 2017–2018 and Census of India 2019. Using population projection as of 1st January 2018 from Census of India 2019

As far as the skill-mix ratio is concerned, the stock data of NHWA suggests nurse-to-doctor ratio as to be 2.02:1 at the all India level, with large-scale variations across states varying from 10.7:1 in Himachal Pradesh and 9.9:1 in Haryana on the higher side to as low as 0.4:1 in Bihar and 0.6:1 in Uttarakhand. The nurse-to-doctor ratio on the basis of the NSSO data, however, is estimated to be 1.7:1 at the all India level with Punjab (7.1:1) and Delhi (4.8:1) on the higher side and states of Bihar, Jammu & Kashmir and Madhya Pradesh having less than 1 nurse per doctor on the lower side (Table 4). Figure 3 presents skill-mix ratio as against density of doctors at the state levels.

**Table 4:** Skill-mix of health workers in different states, 2018

	Nurse/doctor		Traditional medicine including AYUSH/doctor		Allied professional/doctor
State	NSSO	NHWA	NSSO	NHWA	NSSO
Andhra Pradesh	2.7	3.7	0.8	0.2	1.2
Assam	4.4	2.1	0.4	0.1	1.7
Bihar	0.5	0.4	0.7	3.4	0.1
Chhattisgarh	4.9	3	1.3	0.6	1.1
Delhi	4.8	3.4	0.6	0.6	0.6
Gujarat	2.2	2.6	0.8	0.7	2.4
Haryana	3.4	9.9	1.2	2.5	2.6
Himachal Pradesh	6.5	10.7	2.7	3.8	5.9
Jammu and Kashmir	0.6	0	0.5	0.4	0.9
Jharkhand	1.9	1.4	0.2	0.1	0.6
Karnataka	1.7	2.3	0.6	0.4	0.7
Kerala	0.9	5.2	0.6	0.7	0.4
Madhya Pradesh	0.6	4.1	0.4	1.8	0.5
Maharashtra	2.0	1.2	0.6	0.9	1.1
Odisha	3.6	6.1	1.4	0.6	1.4
Punjab	7.1	2.1	1.7	0.3	4.4
Rajasthan	3.8	7.1	1.0	0.4	1.1
Tamil Nadu	2.8	2.6	0.6	0.1	1.0
Telangana	1.8	3.1	0.3	4.2	1.8
Uttar Pradesh	1.3	1.7	0.6	1.1	1.2
Uttarakhand	4.5	0.6	0.6	0.5	0.7
West Bengal	1.0	1.9	0.5	0.6	0.5
India	1.7	2.1	0.6	0.7	0.9

Sources: NHWA 2018 and NSSO 2017–2018

### Estimated skilled health workforce size by 2030

Table 5 depicts the estimated number of skilled health workers (doctors/nurses and midwives) for 2019 through 2030. The base line number for 2019 has been taken from the education adjusted estimates of health workforce from the NSSO 2017–2018 (Table 2). The projected skilled health workforce numbers will rise from current estimates of 1.77 million to 2.65 million in 2030. However, even this will not result in a rise of the skilled health workforce density as the density will be approximately 17.5 per 10,000 population in 2030. There will be a shortfall of approximately 1.13 million skilled health workers to reach 22.8 skilled health workers per 10,000 population. However, if there is a scale-up of nursing supply to approximately 200% growth by 2030, the resultant number of nurses will be 2.02 million in 2030 and the total skilled health workforce number will be 3.45 million in 2030 (22.76 skilled health professionals per 10,000 population).

**Table 5:** Projected skilled health workforce (2019 to 2030)

Year/forecast point	Population in billion (India)	Doctors (in million)	AYUSH (in million)	Nurses (in million)	Projected skilled health workforce (in million)	Skilled health workforce needed to reach 25/10,000 (in million)	Gap (in million)
2019/baseline*	1.369	0.65	0.32	0.80	1.77	3.42	1.65
2025/forecast mid-point	1.452	0.76	0.42	1.04	2.23	3.62	1.40
2030/forecast end-point	1.513	0.93	0.50	1.22	2.65	3.78	1.13

These figures consider adjusted NSSO numbers (workforce numbers adjusted for education qualifications)

*From NSSO estimates

If the NSSO-reported data for health professionals without any adjustment for educational qualifications is considered as the baseline, the projected estimates of skilled health workforce numbers would be 3.03 million and density will be approximately 20.03 per 10,000 population in 2030 at current growth rates. There will be a shortfall of approximately 0.7 million skilled health workers to reach 25 skilled health workers per 10,000 population. The forecasted supply side scenario from 2020 to 2030 is presented in Additional file 1: Appendix Table A-IV.

### Distribution of health workforce by gender and age

**Fig. 4:**
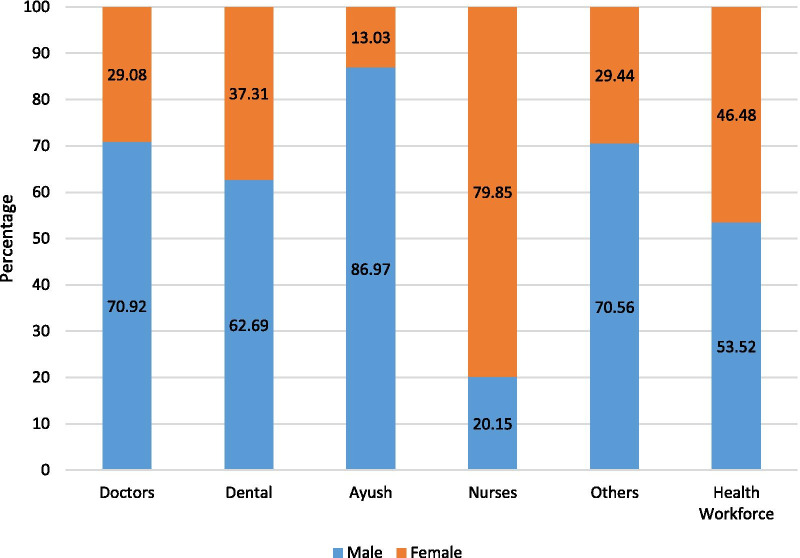
Gender distribution of HRH in India-2018. Source: estimates from NSSO 2017–2018

**Fig. 5:**
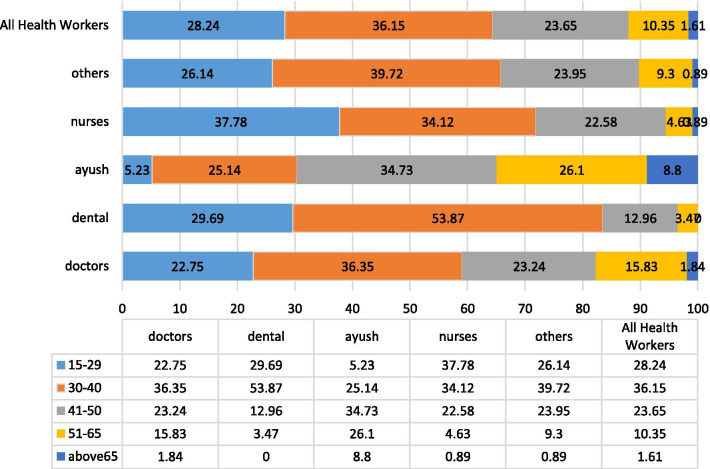
Age distribution of health workforce in India-2018. Source: estimates from NSSO 2017–2018

The gender and age distribution of health workforce (Figs. 4 and 5, respectively) reveals that there is a clear numerical dominance of males in doctors, dental and AYUSH categories, whereas females outnumber male in the nurse’s category. Approximately two-thirds of all health workforce are below age 40 years while more than 25% being in the young age group of below 30 years. Nurses and dentists reflect higher concentration, 38% 30%, respectively, in the younger age group (15–29 years) as compared with doctors (23%) and other health workers. Accordingly, doctors have higher concentration in the older age group of 50 years and above (18%) as against 3% dentists and 5.5% nurses in the same age group.

### Distribution across rural–urban and public–private

The uneven distribution of health workers is also reflected across rural–urban and public–private settings (Additional file 1: Appendix Figure-A-I and Figure A-II). Although rural India constituted approximately 66% of the total population in 2018, only 33% of all health workers are in rural areas. This proportion is a quite lower for dental work force. The proportions of doctor and nurses in rural areas are 27% and 36%, respectively. Further, the bulk of the total health workforce is employed in the private sector. Approximately 60% of inpatient care and 70% of outpatient care in India is provided by private sector [34]. The proportions employed in the private sector: doctors (65%), dentists (89%), AYUSH (93%) and other health workers (67%) are also to a great extent commensurate to the proportion of service delivery.

### Person with medical education but out of labor force

**Fig. 6:**
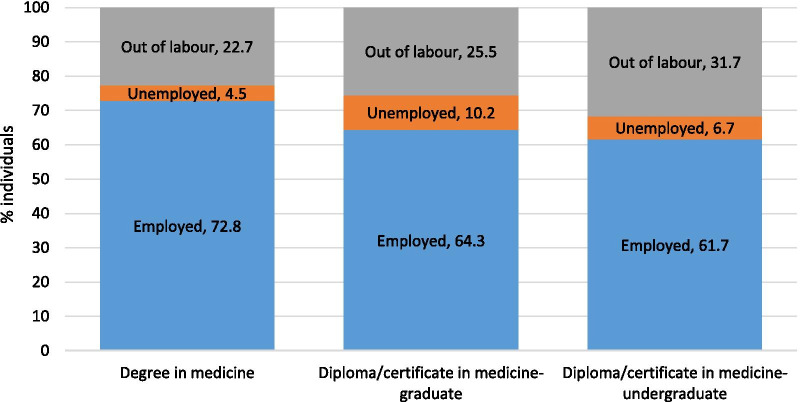
Percentage distribution of individuals with various levels of technical education in medicine as employed, unemployed and out of labor force, 2018. Source: estimates from NSSO 2017–2018

Further a substantial proportion of medically qualified persons are not the part of current health workforce. The estimates from the NSSO indicate that among the individuals with a qualification of degree in medicine (graduate and above), 27% are not active in labor market while approximately 4% are currently unemployed and looking for jobs (Fig. 6). Similarly, among the diploma holders, above or below graduate levels, only 63% reported currently employed.

We also examined the gender and age profile of the persons who have technical education in medicine but are ‘out of labor force’ and noted that female shares an overwhelming proportion (31%) of persons with technical education in medicine but are out of labor force. Proportions of persons with technical education in medicine but out of labor force is higher in the younger and elderly age groups. However, approximately 20% female who are not in the labor force and have technical education in medicine are in the age group of 30–40 years (Additional file 1: Appendix Figure A-III). An overwhelming proportion of these women reported themselves engaged in household work as against joining labor markets.

## Discussions and policy implications

Investment in HRH to improve availability of health workforce has gained increased attention in recent years [2, 5]. In India such investments also have potential to enhance female labor force participation and formalization of labor market [15]. These discussions on enhancing the investment and policy attention to health workforce-related issues has assumed centrality in the presence of the COVID-19 pandemic.

In the present report, we presented different dimensions of HRH in India, along with existing and emerging challenges which need to be addressed for improved availability of health workforce in the country as a whole and at the state levels. We used two nationally representative data sources on health workforce: (i) stock of health workforce from the NHWA 2018 and (ii) National sample survey data (NSSO) 2017–2018 on labor force to identify HRH challenges and areas of investment in HRH in India. Our estimates from the NHWA data are almost similar to the results as reported in a recent WHO report [15]. However, NHWA and NSSO-based estimates in the present study reflect widely varied estimates on the size of health workforce with the NHWA-based estimates significantly higher to the NSSO-based estimates.

Several reasons have been highlighted explaining the difference between the estimates of health professionals from the NHWA data and health workers as reported in the NSSO data [14, 18]. Most of these reasons are related to the fact that a large proportion of the health professionals registered with different councils and associations are not part of the current health workforce in India. One widely discussed reason is the migration of qualified health professionals from India to other developed countries [8, 13, 35, 36].

In addition, there are reasons related to the veracity and updating of the NHWA data. For instance, the NHWA data are collated from different professional councils, which do not maintain a live register and do not require renewing the registration. The information they provide is fraught with non-adjustment of health professionals leaving the workforce because of death, retirement and double counting of workers because they have registered in more than one state [14, 18].

However, one of the most important reasons of this differential estimate is that the NHWA provides total stock of health professionals, but not all of them are active in labor markets. Using NSSO, we reported in this paper that a substantial proportion of medically qualified individuals, overwhelmingly women, is currently not a part of workforce, either because they are currently unemployed but available for work or they do not want to join labor markets. This is particularly amplified for nurses/midwives, for whom the difference between the registered and active workers is the highest. If we apply these proportions (% employed) over the NHWA stock data, we come to pretty close estimates from the two sources.

Despite the differences in estimates of health workforce across the two main sources of information, both the sources indisputably reflect skewed distribution of health workforce across states and inadequate skill-mix ratio.

AYUSH practitioners are recognized health professionals by government of India and they use indigenous system of healthcare. Use of indigenous knowledge in health system is not unique in India. Such system exist in many developing countries including Bangladesh, China and South Africa [37–39] and the Traditional Chinese Medicine was also used as a safeguard against SARS and COVID-19 in China [40]. In India, a large section of population has significant belief in AYUSH system and for many chronic conditions AYUSH is often preferred over modern healthcare by a large proportion of population [41, 42]

Density of health workforce with respect to population is an important indicator of availability of health workforce. Density of allopathic doctors and nurses who are active in labor market are as low as 6.1 and 10.6, respectively, per 10,000 persons (16.7 in total), which is well below the WHO threshold of 44.5 doctors, nurses and midwives per 10,000 population. If we add dentists and AYUSH professionals, the total active health workforce density comes to be approximately 22 per 10,000 persons. The present study clearly reveals that new investment for improving the size of active health workforce is the most important area which needs policy attention in India.

In addition, we also find a sub-optimal skill-mix between doctor and nurse and doctor and allied health professional. Size of traditional medicine practitioners (including AYUSH) in India is quite sizeable. Total number of active AYUSH practitioners is almost 70% of the total number of active allopathic doctors.

However, the number of nurses per doctor is less than 2. This number is lower to 1 if we consider BSc Nursing qualifications. In most OECD countries there are 3–4 nurses per doctors [8]. We find that although total stock of nurses in the country is approximately 3 times number of doctors, a large proportion of nurses are not actually active in labor market. In order to increase nurses’ participation in active health workforce, creating a smooth employment environment for nurses may be another area of policy intervention. There is a need to make balance between densities of doctor and nurse both for a better availability of health professionals and skill-mix. Similarly, doctor/allied health professionals’ ratio is also very poor which needs attention. The Global Strategy report [4] and other similar studies [43] also emphasized creation of enough allied health professionals through improved training and educational infrastructure.

Skewed distribution of health workforce across states and rural–urban setting is yet another area which needs policy attention. Nearly two-thirds of all health workforce in India is concentrated in urban areas leaving rural population either in extreme unmet need of health workers or to avail their services by travelling in urban areas or both. The lop-sided distribution of health workforce is also pronounced across Indian states. Most of the less developed states such as Bihar, Jharkhand, Odisha, Rajasthan, Uttar Pradesh, etc., reflect the acute shortage of health workforce. To understand the reasons of such skewed distribution across states and to understand regional level complexities, a more detailed and deeper study is required.

As far as public–private division of health workforce is concerned, the bulk of doctors’ employment is in private sector while nurses are almost equally distributed across public and private sector. Public sector seems to be sole employer of traditional medical practitioners. These lop-sided distribution of health workers not only creates shortage of trained health workforce in many states and rural areas, but also leads to unequal skill-mix across different types of health workers in different settings. These findings are in conformity with earlier studies [14, 20].

The public sector is also challenged by a high rate of vacancy of sanctioned positions [44]. While the shortage is most pronounced for specialists at Community Health Centres, the shortages are prominently witnessed across the states for various positions. The existing vacancies are attributed to diverse reasons that range from barriers in recruitment, litigations against recruitment processes and premature exits from the system, especially in contractual positions. Filling up existing vacancies in government sector requires urgent policy attention.

An analysis of the health workforce projections suggests that the estimated density of skilled health professionals (doctors, nurses and midwives) per 10,000 population is unlikely to alter from current levels by 2030 if the current rates of growth are sustained. While we are to witness an absolute rise in numbers by 2030, the density of the health workforce is unlikely to change by 2030. AYUSH represents Indian systems of medicine which are predominantly accessed by people of Indian origin, and their inclusion might introduce difficulty in creating comparisons with other countries. Nonetheless, we feel that since there is a significant government emphasis and investment in their training and deployment, as well as them sharing a large clientele in the population, they merit an inclusion in the overall workforce numbers. We have presented the AYUSH numbers as distinct from doctors, but we have included them in the calculation of the overall skilled health worker density.

At the present level of the growth in the supply side, the skill-mix ratio of doctor: nurse is unlikely to alter by 2030. A near 200% growth in the supply side for nurses will improve the doctor: nurse ratio to 1:1.5 by 2030. This will require a further rapid scale-up of nursing programs. The High Level Expert Group report for the Planning Commission in 2012 [45] had suggested a ratio of 1:2:1 for doctor:nurse:ANM for India. For achieving this number of nurses by 2030, simultaneous efforts will have to be undertaken on the demand side of the market as well. The roles for nurses and the functions that are performed by them will need closer attention.

The analysis in this study throws several points for policy interests as follows:

*Expanding the supply side of the health workforce* The expansion of medical educational institutions (medicine, nursing, dentistry, etc.) should be prioritized across geographical regions with a shortage of health workforce and the passed out from these institutions should be encouraged to work in local areas. Thailand represents a good example of effective implementation of rural retention policies for medical doctors [46]

*Growth in the number of nurses in the workforce needs priority attention* The creation of new infrastructure/institutions for nursing may be a medium- to long-term intervention. Also, efforts should be taken to expand the capacity and quality of existing institutions to train the nurses.

*Increasing participation of trained personnel in the workforce *A significant proportion of the trained manpower, especially women, is not present in the workforce. Strategies for re-skilling these graduates and attract them in labor markets should be worked out.

*Balancing the skill-mix* The existing skill-mix is doctor-centric with a lower number of nurses. An emphasis on significantly increasing nursing supply and retaining the nurses in the workforce needs to be evolved at the national level. The specific role of task-shifting and its impact on patient-care and well-being will need greater attention.

*Fast-tracking recruitment and deployment for public health facilities* Improve effectiveness of recruitment processes by walk-in interviews or contractual/flexible norms of engagements to reduce the existing human resource gaps in public sector institutions, particularly at the primary levels.

*Harnessing technology* Covid-19 has highlighted the potential to make more effective use of new and emerging technology to improve the delivery of care, to enable rapid and effective communications, and to improve access to care via e-health and m-health interventions. This is an area where investment in technology and in training the workforce can have dividends.

*Up-skilling programs for less qualified care providers* There is a section of the health workforce which has lower than desirable qualification as reported in the NSSO data. This issue needs deliberation within the Councils and the Ministry of Health at the national level to identify the mechanisms to address the issue. While we do not recommend their formalization in the workforce in the present form, the government can consider up-skilling programs to improve the quality of services and engage them in a range of care giving and non-medical health services.

*Improving HWF information *A significant overhaul and improvement of data on registration of health professionals with live registers of health professionals at the country level is required, with a regular/periodic update and adjustment of the data base. The presence of live registers will replace the reliance on estimates from surveys and give a clearer picture for prompt decision-making and workforce planning for the future, as well as contributing to ongoing quality assurance of the registered professionals.

Implementing the above recommendations will require substantive increase on investment in the health workforce, which will contribute to inclusive economic growth in India.

## Supplementary Information

**Additional file 1: Appendix A-I.** Classification and identification of Health workforce according to NIC and NCO codes. **Appendix A-II.** Percentage distribution of health workforce across states. **Appendix A-III.** State-wise density of the Health workforce in India-2018. **Appendix Table A-IV.** Forecasted number of seats available annually from 2020 to 2030. **Appendix Figure A-I.** Distribution of HWF in India 2018 across rural and urban. **Appendix Figure A-II.** Distribution of HWF in India 2018 across private public settings. **Appendix Figure A-III.** Percentage distribution of male and female with technical education in medicine and out of labor force by age groups

## Data Availability

Data for this study were used from secondary sources. Micro data from the NSSO are available for free in public domain from the official website (http://www.mospi.gov.in/unit-level-data-report-nss-75th-round-july-2017-june-2018-schedule-250social-consumption-health) of the National Sample Survey Office, Ministry of Statistics and Programme Implementation, Government of India.

## Abbreviations

ANM: Auxiliary nurse midwife
AYUSH: Ayurveda, Yoga and Naturopathy, Unani, Siddha and Homeopathy
CBHI: Central Bureau of Health Intelligence
ComHEEG: High-Level Commission on Health Employment and Economic Growth
HRH: Human Resources for Health
LMICs: Lower and middle-income countries
MoHFW: Ministry of Health and Family Welfare
NCO: National Classification of Occupations
NHP: National Health Policy
NHWA: National Health Workforce Account
NIC: National Industrial Classification
NITI Aayog: National Institution for Transforming India
NSSO: National Sample Survey Office
OECD: Organisation for Economic Cooperation and Development
PLFS: Periodic Labour Force Survey
RMP: Registered Medical Practitioner
SDG’s: Sustainable Development Goals
UN: United Nations
WHO: World Health Organization
WPR: Worker population ratio
