# Supplementary material for: Size, composition and distribution of health workforce in India: why, and where to invest?
Source: Hum Resour Health. 2021 Mar 22;19:39. doi: 10.1186/s12960-021-00575-2 (PMC7983088; doi:10.1186/s12960-021-00575-2)
Supplement: Supplementary file 1 — Additional file 1: Appendix A-I. Classification and identification of Health workforce according to NIC and NCO codes. Appendix A-II. Percentage distribution of health workforce across states. Appendix A-III. State-wise density of the Health workforce in India-2018. Appendix Table A-IV. Forecasted number of seats available annually from 2020 to 2030. Appendix Figure A-I. Distribution of HWF in India 2018 across rural and urban. Appendix Figure A-II. Distribution of HWF in India 2018 across private public settings. Appendix Figure A-III. Percentage distribution of male and female with technical education in medicine and out of labor force by age groups [file 12960_2021_575_MOESM1_ESM.docx]

**Supplementary File**

**Appendix A-I. Classification and identification of Health workforce according to NIC and NCO codes.**

National Industry Classification (NIC) 2008 uses five-digit code for classification and identification of all economic activities including health. Health workers are identified by using the codes 86100 to 86909. In addition, workers in retail trade of pharmacy were identified by the code 47721. Health workers with NIC and NCO codes are given in table below.

National Classification of Occupations (NCO) 2004 is a hierarchical skills-based classification of occupation which consists of 10 divisions (one-digit code); 30 sub-divisions (two-digit code); 116 groups (three-digit code); 439 families (four-digit code) and 2945 occupations (six-digit code). We have used three digit codes for identification in our study as NSSO provides three-digit classification of the occupations. The health workers with their respective NCO identification codes are given in table below.

NIC and NCO Codes for identifying health workers:

| NIC CODES 2008 | NCO CODES 2004 | HEALTH WORKFORCE |
| --- | --- | --- |
| 86100 | 222 | Doctors, Dentists, AYUSH |
| 86201 | 222 | Doctors |
| 86202 | 222 | Dentists |
| 86901 - 86903 | 222 | AYUSH |
| 86904 - 86909 | 222 | Doctors (Paramedics) |
| 87100 | 223 | Nurses |
| 86100 - 86909 | 323 | Mid- wives |
| 87100 - 87900 | 222,223, 322, 323 | Resident Nurses |
| 86100 – 86909, 87100 - 87900 | 322,324 | Allied personnel |
| 47721 | 211, 221, 323, 324 | Pharmacists |

Appendix A-II. Percentage distribution of health workfoce across states

|  | NHWA (in thousands) | | | | | NSSO (in thousands) | | | | | |
| --- | --- | --- | --- | --- | --- | --- | --- | --- | --- | --- | --- |
| States | Allopathic doctors | Nurses | Traditional including AYUSH | Dental | Pharmacist | Allopathic doctors | Nurses | Traditional including AYUSH | Dental | Allied | Pharmacist |
| Andhra Pradesh | 100.6 | 371.1 | 22 | 21.4 | 51.8 | 34.7 | 92.9 | 29.5 | 6.8 | 40.8 | 13.3 |
| Assam | 23.9 | 50.3 | 2.2 | 2.7 | 12.3 | 11.7 | 51.2 | 4.7 | 0.6 | 19.7 | 14.5 |
| Bihar | 40.6 | 18 | 136.5 | 8.6 | 24.3 | 56.4 | 26.0 | 39.8 | 2.9 | 4.2 | 12.0 |
| Chhattisgarh | 8.8 | 26.4 | 5.6 | 3.8 | 17.3 | 8.3 | 40.3 | 10.6 | 2.1 | 9.2 | 11.8 |
| Delhi | 21.4 | 71.9 | 12.2 | 15.3 | 30.9 | 12.6 | 60.2 | 7.8 | 13.1 | 8.0 | 0.0 |
| Gujarat | 66.9 | 171.7 | 50 | 15 | 69.5 | 31.6 | 69.6 | 25.7 | 11.9 | 74.4 | 10.5 |
| Haryana | 5.7 | 57 | 14.1 | 8.8 | 35.4 | 11.0 | 37.3 | 13.0 | 4.0 | 28.2 | 6.9 |
| Himachal Pradesh | 3.1 | 32.6 | 11.6 | 2.4 | 9.1 | 1.1 | 7.2 | 2.9 | 3.8 | 6.5 | 0.0 |
| Jammu & Kashmir | 15 | 0 | 6.1 | 4 | 0 | 19.0 | 10.9 | 8.8 | 4.7 | 17.5 | 6.1 |
| Jharkhand | 5.8 | 8.1 | 0.8 | 0.1 | 3.5 | 11.7 | 22.2 | 2.1 | 2.9 | 7.3 | 2.5 |
| Karnataka | 122.9 | 285.7 | 48.3 | 43.9 | 60.5 | 37.2 | 61.4 | 22.8 | 19.5 | 26.4 | 19.8 |
| Kerala | 59.4 | 306.3 | 41.6 | 21.3 | 68.4 | 88.9 | 81.8 | 56.6 | 7.0 | 31.8 | 16.6 |
| Madhya Pradesh | 38.2 | 158.4 | 67.1 | 8 | 59.2 | 67.9 | 42.3 | 25.2 | 7.4 | 34.8 | 17.6 |
| Maharashtra | 173.4 | 210.3 | 153.1 | 40.5 | 250 | 60.0 | 122.8 | 37.7 | 50.0 | 65.7 | 10.7 |
| Odisha | 22.5 | 137.7 | 14.7 | 2 | 34.1 | 9.1 | 33.1 | 12.6 | 2.9 | 12.5 | 9.0 |
| Punjab | 48.4 | 99.7 | 16 | 15.6 | 49.1 | 6.6 | 47.1 | 11.2 | 1.9 | 29.4 | 16.9 |
| Rajasthan | 43.4 | 308.9 | 18.8 | 6.6 | 51.5 | 19.3 | 74.1 | 18.5 | 3.5 | 21.9 | 26.0 |
| Tamil Nadu | 135.5 | 351.5 | 18.8 | 22.8 | 87.9 | 54.0 | 153.7 | 34.9 | 13.8 | 55.8 | 15.9 |
| Telangana | 4.9 | 15.3 | 20.9 | 1 | 64.9 | 31.5 | 55.6 | 9.2 | 8.2 | 57.1 | 11.7 |
| Uttar Pradesh | 77.5 | 135 | 85.5 | 18.5 | 91.3 | 109.9 | 139.5 | 62.1 | 10.3 | 132.3 | 19.4 |
| Uttarakhand | 8.6 | 5 | 4.1 | 1.2 | 17.3 | 5.9 | 26.8 | 3.3 | 0.6 | 4.0 | 1.0 |
| West Bengal | 72 | 136.1 | 46.9 | 5.7 | 89.6 | 91.2 | 94.4 | 48.5 | 2.1 | 49.4 | 7.7 |
| NE States | 4.3 | 40.2 | 1.4 | 1.1 | 9.7 | 15.0 | 37.2 | 1.5 | 2.0 | 9.7 | 0.4 |

Source: Estimates from NSSO 2017-18

Appendex A-III. State-wise density of the Health workforce in India-2018

|  | Doctors | | Nurses | | Traditional | | Allied |
| --- | --- | --- | --- | --- | --- | --- | --- |
| State | NSSO | NHWA | NSSO | NHWA | NSSO | NHWA | NSSO |
| Delhi | 6.50 | 8.98 | 30.97 | 30.2 | 4.00 | 5.12 | 4.11 |
| Haryana | 3.88 | 1.97 | 13.19 | 19.67 | 4.61 | 4.87 | 9.98 |
| Himachal | 1.52 | 4.18 | 9.93 | 44.61 | 4.06 | 15.9 | 8.94 |
| J&K | 14.21 | 11.67 | 8.12 | 0 | 6.57 | 4.76 | 13.06 |
| Punjab | 2.24 | 16.06 | 15.90 | 33.12 | 3.78 | 5.31 | 9.92 |
| Rajasthan | 2.53 | 5.65 | 9.72 | 40.24 | 2.43 | 2.45 | 2.87 |
| Uttarakhand | 5.36 | 7.74 | 24.38 | 4.51 | 3.01 | 3.66 | 3.64 |
| Assam | 3.44 | 7.06 | 15.09 | 14.86 | 1.39 | 0.64 | 5.79 |
| NE States | 9.60 | 2.82 | 23.76 | 26.45 | 0.96 | 0.89 | 6.18 |
| Chhattisgarh | 2.93 | 3.24 | 14.21 | 9.75 | 3.75 | 2.07 | 3.24 |
| Madhya Pradesh | 8.37 | 4.65 | 5.22 | 19.28 | 3.11 | 8.17 | 4.30 |
| Uttar Pradesh | 4.95 | 3.35 | 6.28 | 5.83 | 2.80 | 3.69 | 5.96 |
| Bihar | 4.79 | 3.75 | 2.21 | 1.66 | 3.38 | 12.59 | 0.35 |
| Jharkhand | 3.17 | 1.65 | 6.03 | 2.29 | 0.56 | 0.23 | 1.98 |
| West Bengal | 9.47 | 7.45 | 9.80 | 14.08 | 5.03 | 4.86 | 5.13 |
| Odisha | 2.10 | 5.15 | 7.62 | 31.47 | 2.89 | 3.36 | 2.88 |
| Maharashtra | 4.96 | 13.79 | 10.14 | 16.73 | 3.12 | 12.18 | 5.43 |
| Gujarat | 4.71 | 10.22 | 10.38 | 26.2 | 3.83 | 7.63 | 11.10 |
| Andhra Pradesh | 6.68 | 19 | 17.89 | 70.1 | 5.68 | 4.15 | 7.86 |
| Karnataka | 5.70 | 19.08 | 9.40 | 44.35 | 3.49 | 7.5 | 4.04 |
| Kerala | 25.43 | 16.3 | 23.40 | 84.11 | 16.20 | 11.43 | 9.11 |
| Tamil Nadu | 7.17 | 19.18 | 20.40 | 49.78 | 4.64 | 2.66 | 7.40 |
| Telangana | 8.51 | 1.3 | 15.04 | 4.03 | 2.49 | 5.5 | 15.44 |
| UTs | 15.62 | 0 | 28.30 | 0 | 7.16 | 0.29 | 22.54 |

Source: Estimates from NSSO 2017-18

Appendix Table A-IV: Forecasted number of seats available annually from 2020 to 2030

| **Qualification** | **2020** | **2021** | **2022** | **2023** | **2024** | **2025** | **2026** | **2027** | **2028** | **2029** | **2030** |
| --- | --- | --- | --- | --- | --- | --- | --- | --- | --- | --- | --- |
| Allopathic Doctors ^+^ | 57138 | 61424 | 65710 | 69997 | 74283 | 78569 | 82855 | 87141 | 91428 | 95714 | 100000 |
| Dentists^ | 27439 | 27929 | 28419 | 28909 | 29399 | 29889 | 30379 | 30869 | 31359 | 31849 | 32339 |
| AYUSH* | 40881 | 41699 | 42516 | 43334 | 44151 | 44969 | 45787 | 46604 | 47422 | 48240 | 49057 |
| Nurses** | 98229 | 100194 | 102158 | 104123 | 106087 | 108052 | 110017 | 111981 | 113946 | 115910 | 117875 |
| Pharmacists* | 47646 | 48497 | 49347 | 50198 | 51049 | 51900 | 52751 | 53602 | 54452 | 55303 | 56154 |
| ANM** | 56268 | 57273 | 58277 | 59282 | 60287 | 61292 | 62296 | 63301 | 64306 | 65311 | 66316 |

*Data Sources: Annual seat capacity (2019): +MCI data, ^Dental Council of India – 2019-20 session (as on 10 Sept 2019), *NHP 2018, ** Indian Nursing Council 2017-18 Annual Report (as on 31st March, 2018), #NIAHS report, Public Health Foundation of India*

*For allopathic doctors we used a number of 100,000 to be the size of the supply side in 2030.*

Appendix Figure A-I. Distribution of HWF in India 2018 across rural and urban

Source: NSSO 2017-18

Appendix Figure A-II. Distribution of HWF in India 2018 across private public settings

Source: NSSO 2017-18

Appendix Figure A-III: Percentage distribution of male and female with technical education in medicine and out of labour force by age groups

Source: Estimates from NSSO 2017-18
